# Resistance to targeted therapies in acute myeloid leukemia

**DOI:** 10.1007/s10585-022-10189-0

**Published:** 2022-11-01

**Authors:** Rabea Mecklenbrauck, Michael Heuser

**Affiliations:** Department of Hematology, Hemostasis, Oncology and Stem Cell Transplantation Hannover Medical School, Carl-Neuberg-Str. 1, 30625 Hannover, Germany

**Keywords:** Primary resistance, FLT3 inhibitors, IDH inhibitors, Gemtuzumab-ozogamicin, Venetoclax

## Abstract

The introduction of new targeted therapies to the treatment algorithm of acute myeloid leukemia (AML) offers new opportunities, but also presents new challenges. Patients diagnosed with AML receiving targeted therapies as part of lower intensity regimens will relapse inevitably due to primary or secondary resistance mechanisms. In this review, we summarize the current knowledge on the main mechanisms of resistance to targeted therapies in AML. Resistance to FLT3 inhibitors is mainly mediated by on target mutations and dysregulation of downstream pathways. Switching the FLT3 inhibitor has a potential therapeutic benefit. During treatment with IDH inhibitors resistance can develop due to aberrant cell metabolism or secondary site IDH mutations. As a unique resistance mechanism the mutated IDH isotype may switch from IDH1 to IDH2 or vice versa. Resistance to gemtuzumab-ozogamicin is determined by the CD33 isotype and the degradation of the cytotoxin. The main mechanisms of resistance to venetoclax are the dysregulation of alternative pathways especially the upregulation of the BCL-2-analogues MCL-1 and BCL-XL or the induction of an aberrant cell metabolism. The introduction of therapies targeting immune processes will lead to new forms of therapy resistance. Knowing those mechanisms will help to develop strategies that can overcome resistance to treatment.

Between 2017 and 2022, no less than ten new drugs have been approved by the FDA for AML-treatment, which has significantly changed the therapeutic landscape previously dominated by 7 + 3 chemotherapy and hypomethylating agents. Several of these drugs are directed against a specific target in the leukemic cell and allow an individualized therapeutic approach. However, with the emergence of new targeted agents, new resistance mechanisms have been found. The clinical impact of the development of resistance on patients’ outcome is currently unclear and no concepts are available yet whether and how patient monitoring and treatment management should be adapted to emerging treatment resistance.

In this review, we will summarize available evidence on resistance mechanisms to targeted therapies in AML and discuss potential consequences for clinical management of patients and for translational research to develop new strategies that may overcome drug resistance.

## Resistance to FLT3 inhibitors

FLT3 (Fms related receptor tyrosine kinase 3) is a transmembrane kinase located in the cell membrane and the membrane of the endoplasmic reticulum. Binding of the FLT3 ligand (FL) to FLT3 on its extramembraneous binding site leads to the dimerization of FLT3 at the juxtamembrane domain. The phosphorylation of its juxtamembrane domain allows for the binding of substrates which are activated by phosphorylation of tyrosine [1–3]. The activation signal is passed on to the nucleus through several downstream signalling pathways such as RAS/MAPK, PI3K or JAK/STAT, thereby enhancing cell proliferation [2, 4]. *FLT3*-ITD mutations can be found in 25–35% of adult AML patients [5, 6]. A distinct prognostic impact of *FLT3*-ITD mutations has been described for low and high allelic burden depending on the *NPM1* co-mutation [7, 8]. *FLT3*-TKD mutations on the other hand have no known prognostic impact on its own but offer a therapeutic target [9]. *FLT3*-TKD mutations can be found in about 7% of adult AML-patients [6, 10]. FLT3 inhibitors can be classified into type I and type II inhibitors. Type I inhibitors bind to the ATP-binding site, making them more specific for FLT3 and active for *FLT3*-ITD as well as TKD-mutations [11]. Type I inhibitors currently approved by the FDA are gilteritinib and midostaurin, while crenolanib is evaluated in clinical trials. The type I inhibitor midostaurin is approved for combination therapy with induction and consolidation chemotherapy in patients with *FLT3-*ITD or TKD mutations [12]. In the Ratify trial midostaurin prolonged survival independently of the mutational burden of *FLT3* or the co-mutation of *NPM1* [13]. Gilteritinib is used as monotherapy in relapsed or refractory *FLT3*-mutated AML patients, where it was shown to prolong survival compared to conventional chemotherapy [14].

Type II inhibitors bind to the hydrophobic area next to the ATP-binding site [15]. They are only effective with *FLT3*-ITD mutations but are also cross-inhibiting other receptor tyrosine kinases [15]. The most commonly used type II inhibitor is sorafenib. Sorafenib is used as maintenance treatment after allogeneic hematopoietic stem cell transplantation (alloHSCT) and prolongs survival in *FLT3-*mutated patients in this setting [16].

Quizartinib, another type II inhibitor, was recently shown to significantly prolong survival in patients with FLT3-ITD positive relapsed or refractory AML in comparison to placebo [17].

### Resistance to type I inhibitors

The addition of midostaurin to conventional chemotherapy significantly improves survival of *FLT3*-mutated patients in all risk groups including older patients between 60 and 70 years of age [13, 18]. In the Ratify trial, it was found that about half of the patients lost the FLT3 mutation at the time of relapse and acquired mutations in alternative pathways such as the MAPK-pathway, 11% showed an expansion of new FLT3 clones [19]. The F691L gatekeeper mutation confers resistance to midostaurin [20]. The missense mutation N676K also induces resistance to midostaurin in patients [21]. Additionally, a subgroup analysis of the Ratify trial indicated that an insertion in the transmembrane kinase domain is associated with an unfavourable outcome even when adding midostaurin to chemotherapy [22].

Resistance to gilteritinib is mainly mediated by acquired mutations in *FLT3* itself or new mutations in other oncogenes. McMahon et al. analysed samples of patients who relapsed after gilteritinib monotherapy and found mutations in the MAPK/RAS-pathway in one third of the patients [23]. Twelve percent of the patients developed a *FLT3*-F691L mutation but these two mechanisms never coincided [23].

### Resistance to type II inhibitors

Missense mutations in *FLT3* also play a potential role in resistance to type II inhibitors. The on-target mutation D835Y changes the conformation of the binding site, thereby conveying resistance to all type II inhibitors, whereas type I inhibitors with their different binding site are still effective [24]. Other mutations at amino acids F691, D835 and Y842 lead to decreased effectiveness of therapy with type II inhibitors [25–28] (Table 1).

**Table 1:** Overview of on-target mutations conferring resistance to FLT3 inhibition

FLT3 inhibitor	Mutations mediating resistance to FLT3 inhibitors	References
Crenolanib	K429E (extramembrane domain) F691L (gatekeeper residue)	[29, 30]
Midostaurin	F691L (gatekeeper residue) N676K (tyrosine kinase domain I)	[19, 21]
Gilteritinib	F691L (gatekeeper residue)	[23, 31–33]
Quizartinib	D835Y/V/F (activation loop) Y842C/H (activation loop) F691L (gatekeeper residue)	[34, 35]
Sorafenib	D835Y/V (activation loop) Y842C/H (activation loop) F691L (gatekeeper residue)	[20, 25, 28, 36, 37]

The activation loop includes sites for phosphorylation, while the gatekeeper residue controls binding of small molecules to the kinase. Most notably, the F691L mutation in the gatekeeper residue of FLT3 leads to resistance against all FLT3 inhibitors used in clinical practice

In contrast to type I inhibitors, acquired mutations in the MAPK/RAS-pathway rarely confer resistance to type II inhibitors, as the latter cross-inhibit several other components of the MAPK/RAS pathway. Table 1 gives an overview of the most common missense mutations that mediate resistance to FLT3 inhibitors.

### Common resistance mechanisms

One main problem in AML treatment is the constant clonal evolution. Frequently, the blasts at relapse bear a different molecular pattern than at diagnosis. Loss of *FLT3* mutations has been observed even without the selection pressure exerted by the use of FLT3 inhibitors [38, 39]. Additionally, primary resistance can be observed in approximately 30% of patients [40]. It is important to note that *FLT3* mutated cells are usually mutated in one allele only and therefore may express unmutated *FLT3* as well. Because the FL primarily binds to wildtype FLT3, it activates downstream pathways the stronger the more wildtype FLT3 is expressed [41, 42]. Although this mechanism does not cause complete resistance, it can be an explanation that depending on the *FLT3* wt/mut ratio the effect of FLT3 inhibitors might differ, as the activation of downstream pathways via FL is more effective. Additionally, the level of FL increases in chemotherapy induced aplasia and a rising FL expression has been associated with a worse outcome [42, 43].

Soluble factors from stromal cells that activate downstream pathways of FLT3 are able to overcome the effect of FLT3 inhibitors. For example, FGF2 is able to activate the RAS/MAPK pathway independently of FLT3 in vitro and increased levels of FGF2 can be found in patients who relapsed under treatment with FLT3 inhibitors [44]. Although this effect has only been examined regarding resistance to quizartinib, it may be an universal mechanism. Similarly, upregulation of the chemokine CXCL12, which is secreted into the bone marrow stroma by osteoblasts, is able to induce proliferation independently of FLT3 in vitro and in mouse models [45, 46]. Interestingly, the acquisition of different *JAK2*-mutations was associated with FLT3 inhibitor-resistance and the non-canonical *JAK2* V658F mutation was able to activate CSF2RB and circumvent FLT3-dependency in vitro [47]. Upregulation of the CCL5-CCR5-pathway, which is known to play a role in therapy resistance in other malignant diseases as well [48], also mediated FLT3 inhibitor resistance as downstream pathways such as RAS, AKT and STAT5 are upregulated independently of FLT3 activation [49]. Upregulation of STAT5 stands out as a key element of FLT3 inhibitor-resistance. It upregulates PIM1 independently of FLT3 and thereby circumvents FLT3-dependency [50]. Furthermore, STAT5 upregulates AXL-expression in vitro in primary AML-blasts via activation from cytokines from the bone marrow environment like TPO or GM-CSF, which conveys FLT3 inhibitor-resistance by activating PI3K and RAS/RAF [51, 52]. However, AXL can also be activated by hypoxia via HIF-1α independently of STAT5 [52]. As gilteritinib inhibits AXL as well as FLT3, it may still be effective in patients after activation of AXL via the STAT5-pathway or HIF-1α [31]. Although the understanding of these pathway modifications is still restricted to in vitro and mouse models, they may also be important in patients.

An increased intracellular pH is known to enhance cell proliferation and reduce apoptosis. An increase in pH via activation of the Na/H+ ion channel via tescalcin (*TESC*) overexpression leads to FLT3 inhibitor resistance in vitro in cell lines as well as in primary AML cells [53].

FLT3 inhibitors are metabolized by CYP3A4. Therefore, overexpression of this enzyme leads to increased drug degradation [54]. Similarly, high expression levels of the P-glycoprotein Pgp efflux pumps decrease the effectiveness of FLT3 inhibitor-therapy [55]. The efficacy of FLT3 inhibitors can also be reduced by increased binding to plasma proteins at least in vitro. The only FLT3 inhibitor which does not seem to be affected by binding to plasma proteins is gilteritinib [56].

**Fig. 1:**
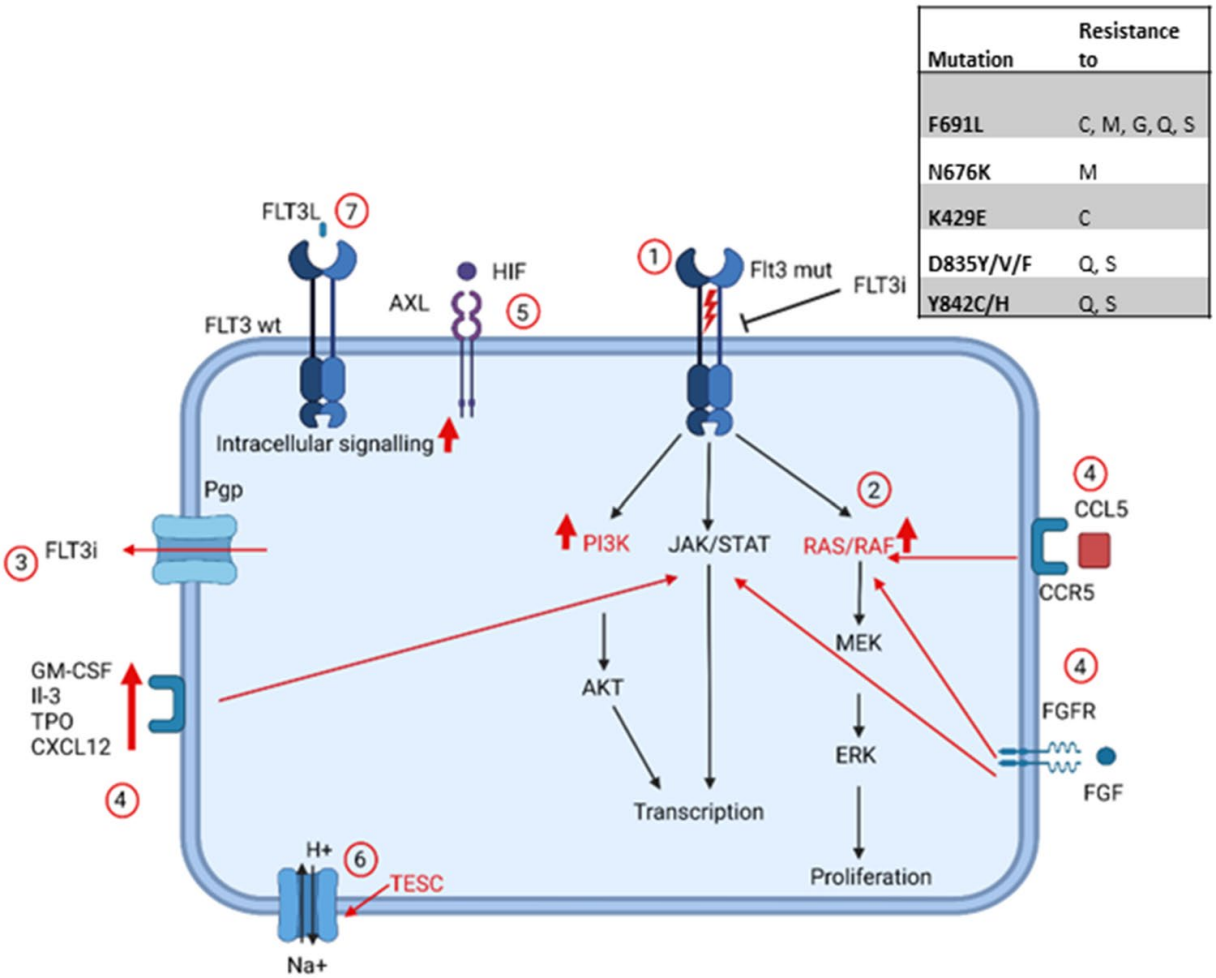
Overview of resistance mechanisms to FLT3 inhibitors. 1: On-target mutations; 2: Activation of downstream pathways; 3: Increased drug elimination; 4: Upregulation of soluble factors to activate downstream pathways; 5: Activation of AXL-pathways; 6: Increase in intracellular pH 7: Upregulation of FLT3 ligand (FL). (Created with BioRender.com) *C* crenolanib, *G* gilteritinib, *M* midostaurin, *Q* quizartinib, *S* sorafenib, *TESC* tescalcin, *FLT3i* Flt3 inhibitor

The currently known resistance mechanisms against FLT3 inhibitors are summarized in Fig. 1.

As more and more patients receive FLT3 inhibitors as frontline therapy, it is important to address the question of efficacy of sequential application of different FLT3 inhibitors. A retrospective analysis of patients relapsing after front line therapy with FLT3 inhibitor-based regimens compared the rates of CR and CRi and highlighted that with each subsequent use of a new FLT3 inhibitor the percentage of patients responding decreased [57]. One obvious reason may be the selection of FLT3-inhibitor-resistant clones independently of the exact binding site of the FLT3 inhibitor such as upregulation of downstream pathways. However, with over 30% achieving CRc with the second FLT3 inhibitor and 25% with the third, the clinical effect is still relevant and may be increased by using combination therapies [57].

## Resistance to IDH inhibitors

Isocitrate dehydrogenase 1 and 2 (IDH1 and IDH2) are vital for the energy metabolism and biosynthesis in the cell as they catalyse the synthesis of α-ketoglutarate from isocitrate as part of the Krebs-Cycle. Mutations in either of these enzymes lead to increased production of R-2-hydroxyglutarate, which inhibits cellular differentiation [58, 59]. In the context of AML, mutations of *IDH1* are found in 6–7% of AML patients while *IDH2* mutations are found in approximately 15% of AML patients [60]. Ivosidenib as IDH1 inhibitor and enasidenib as IDH2 inhibitor are approved by the FDA for monotherapy in patients with relapsed/refractory AML and a proven mutation in the respective enzyme inducing CR in approximately 20% of patients [61, 62]. In 2019, the approval of ivosidenib was broadened to allow first line therapy as a single agent for unfit patients with *IDH1*-mutation. For the IDH1 inhibitor ivosidenib the combination with azacitidine has been shown to be superior to azacitidine monotherapy, inducing CR in 47% vs. 15% in newly diagnosed AML patients, which led to approval by the FDA in May 2022 [63].

The common mutation sites in *IDH1* are R132 [64, 65] and in *IDH2* R140 and R172 [58, 66].

Regarding primary resistance, Wang et al. described that cells with a stem cell-like gene expression profile show a poor response to treatment with IDH inhibitors [67]. This phenotype may be at least partly induced by downregulation of TET2 leading to DNA hypermethylation, which is characteristic for leukemic stem cells (LSC) [68]. Lower response rates to treatment with enasidenib have been associated with *NRAS* mutations [69]. It was also highlighted that at relapse after treatment with IDH inhibitors dominant clones within the blast population were likely to show mutations in *RUNX1* or *NRAS* as well as *FLT3* [69, 70]. It was pointed out as well that one mutation alone may not be sufficient to induce therapy resistance [71].

Newly acquired missense mutations within IDH1 or IDH2 are another central resistance mechanism against IDH1/2 inhibitors. Intlekofer et al. analysed two patients who relapsed under treatment with IDH2 inhibitors by acquiring additional mutations. Of note, the newly acquired mutations in these cases were located on the allele which did not carry the initial R140Q mutation. Both Q316E and I319M mutations inhibit the binding of enasidenib and are pathogenic only in combination with the R140Q mutation. In vivo these mutations have only been identified on the allele not bearing the initial IDH2 mutation but in vitro experiments hint that the acquisition of these mutations on the same allele would also confer resistance [72]. For IDH1 inhibitors the additional acquisition of a S280F or H315D mutation leads to therapy resistance due to changes to the substrate or NADPH binding site [71]. R119P, G131A, D279N and G289D mutations prevent binding due to steric interference [71].

A unique mechanism of resistance to IDH inhibitors is the switching of the mutated *IDH* isotype, e.g. from *IDH1* to *IDH2* or vice versa. This new isotype is either a completely new clone or the expansion of a pre-existing clone [73]. The use of IDH inhibitors, which are able to inhibit both IDH1 and IDH2 may be a strategy to overcome resistance mediated by this isotype switch. Co-targeting agents currently being investigated in AML and glioma are vorasidenib (AG-881) [74] and Ly3410738 [75].

Given its prominent role in energy metabolism, it is not surprising that therapy resistance to IDH inhibitors has been linked to changes in energy metabolism. R-2-hydroxyglutarate leads to an increase in fatty acid oxidation and oxidative phosphorylation via CEBPα. Interestingly, treatment with IDH inhibitors does not diminish fatty acid oxidation (FAO) as it is maintained via PGC1a and its associated pathways in vitro [76]. Upon relapse under IDH inhibitor treatment patients exhibit genetic changes associated with high activation levels of oxidative phosphorylation, which may be a resistance mechanism independent of acquired mutations in the IDH enzymes [76].

One way to improve the response to IDH inhibition may be the combination with hypomethylating agents or standard chemotherapy. In the AGILE-study the combination of ivosidenib with azacitidine induced responses in patients who harboured co-mutations in *NRAS*, *KRAS* and *PTPN11,* who reportedly do not respond as well to ivosidenib monotherapy [77].

**Fig. 2:**
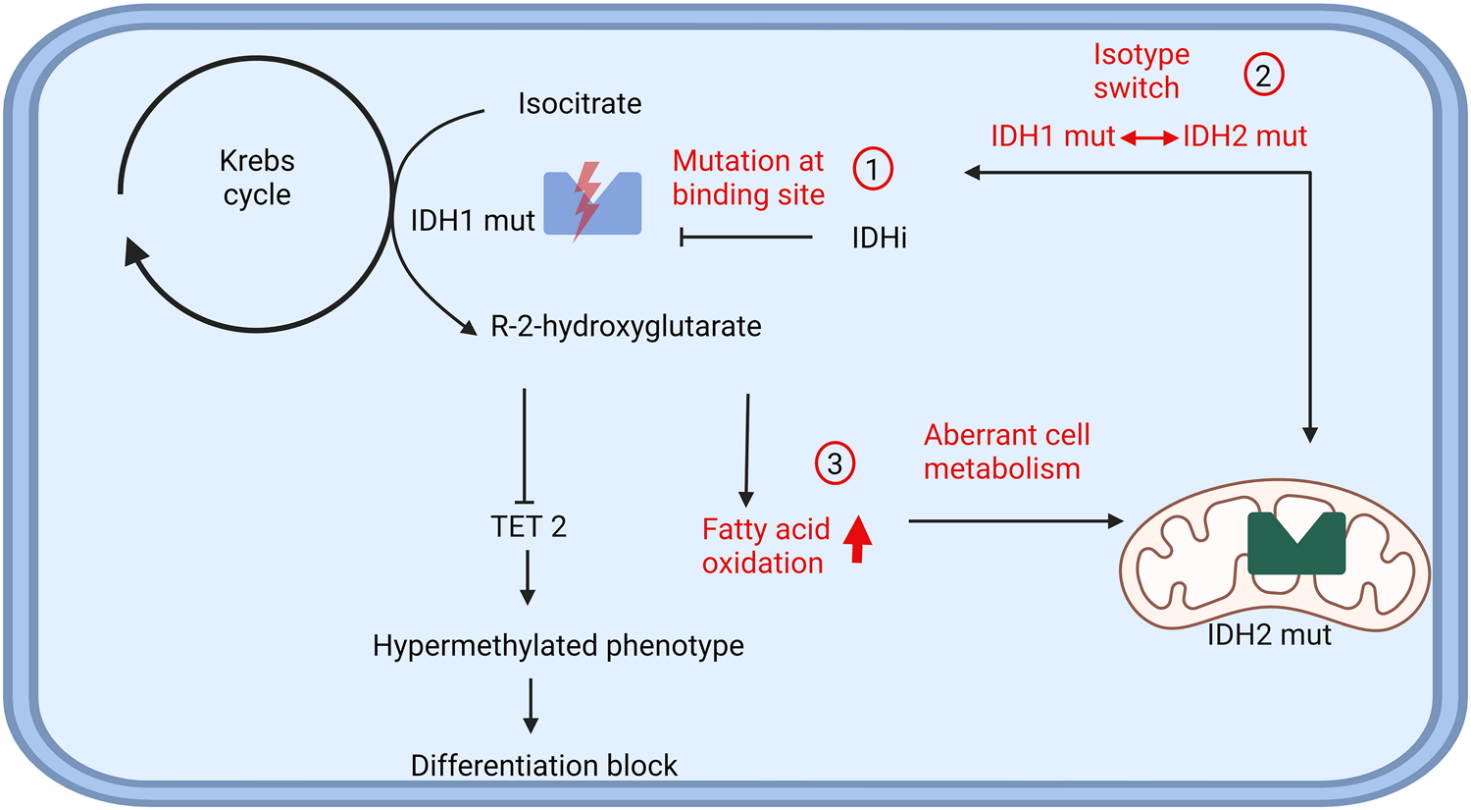
Overview of resistance mechanisms to IDH inhibitors. Treatment with IDH inhibitors mainly blocks the synthesis of the oncometabolite R-2-hydroxyglutarate, which inhibits hypermethylation and induces differentiation of the cell. The upregulation of fatty acid oxidation is not inhibited. The main resistance mechanisms are 1: Mutations at binding site; 2: Isotype switch; 3: Aberrant regulation of cell metabolism. (Created with BioRender.com)

Based on our current understanding resistance to IDH inhibition is thus conveyed by on-target mutations, isotype switching and changes in cell metabolism (Fig. 2).

## Resistance to gemutzumab-ozogamicin (GO)

Gemtuzumab-ozogamicin is a drug-antibody conjugate consisting of an anti-CD33 antibody linked with the cytotoxic drug calicheamicin. Upon internalisation calicheamicin leads to DNA-damage and subsequent apoptosis [78]. It is approved for induction therapy in patients with favourable or intermediate risk CD33 + AML in combination with conventional chemotherapy [79].

Primary resistance to GO is not associated with CD33-expression levels but rather polymorphisms in the gene encoding CD33. This mechanism of primary resistance was firstly described by Lamba et al. in a cohort of pediatric patients treated with standard chemotherapy and GO. The consequence of the polymorphism rs12459419 is an exchange of alanin to valin (C to T) in CD33 [80, 81]. This change leads to a different configuration of the antibody binding-site in CD33. All patients who were homozygous for this polymorphism showed lower response rates than those who were heterozygous [81]. In a larger cohort of patients the mean incidence of each genotype was 51% for CC, 39% for CT and 10% for TT [80]. However, the predictive impact of this polymorphism was variable in different cohorts. Two studies reported no difference in adult cohorts [82, 83], whereas in patients with *NPM1* mutations the polymorphism predicted therapy response [84]. Another polymorphism of CD33, rs35112940, leads to a conformational change in the immunoreception tyrosine-based inhibitory motif. This form of CD33 is less likely to be internalized, thus reducing the cytotoxic effect of GO [85]. However, the prognostic effect of this polymorphism is unknown. So far, it has not been evaluated prospectively, whether CD33 polymorphisms can be used as a predictive marker for GO treatment.

The polymorphism rs1045642 in the gene *ABCB1* encoding P-glycoprotein (Pgp) (C to T) also has predictive value for response to GO. This polymorphism has been associated with inferior response rates to GO in patients who are homozygous for CC [86].

Another mechanism of therapy resistance is associated with an MDR-phenotype and increased expression of Pgp*.* Early work by Walter et al. analysed the outcome of patients treated with GO monotherapy after AML relapse. Higher expression levels of *ABCB1* correlated with a poor response to treatment with GO [87]. However, it is unclear whether this is a mechanism of primary resistance or if *ABCB1* expression is upregulated in response to treatment. Furthermore, this mechanism is not specific for response to GO, but rather shows that *ABCB1* expression is a marker for poor response to chemotherapy in general.

In addition to increased drug efflux, leukemic cells are able to increase CD33 degradation. The vital protein in this mechanism is SOCS3, which binds to phosphorylated CD33 and leads to internalisation and degradation of CD33 [88, 89]. Low levels of promoter methylation of *SOCS3* and therefore increased expression levels correlated with lower response rates to GO treatment as mono- or combination therapy [90].

**Fig. 3:**
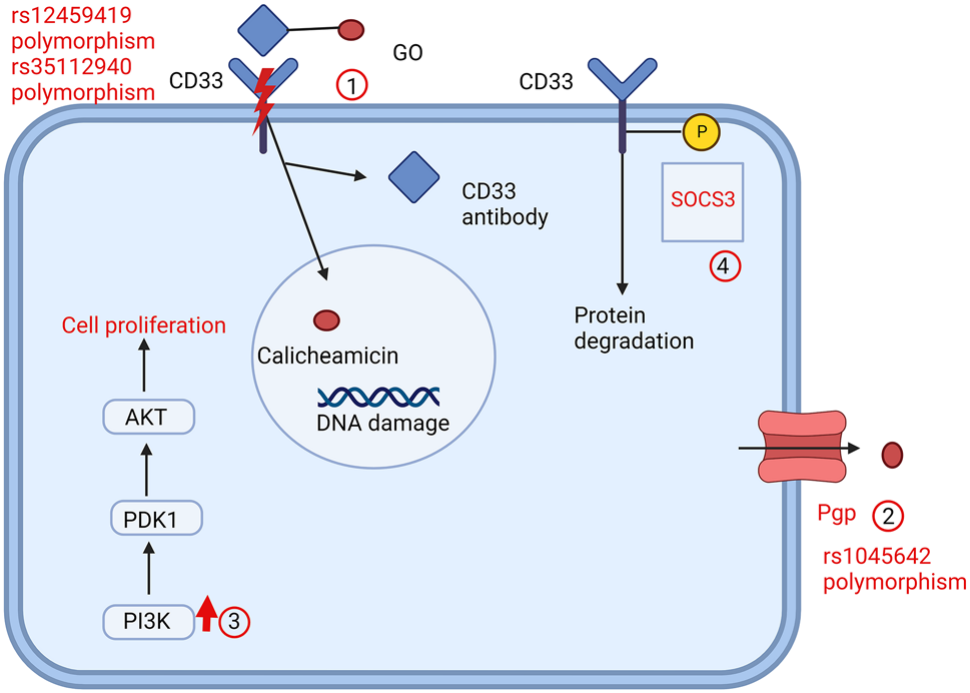
Overview of resistance mechanisms to GO. GO binds to CD33 and is internalized. Inside the cell, calicheamicin dissociates from the CD33-antibody and induces DNA-damage. Main mechanisms of resistance: 1: CD33 polymorphisms; 2: Increased drug degradation; 3: Upregulation of downstream pathways; 4: Phosphorylation of CD33 by SOCS3. (Created with BioRender.com)

Acquired resistance to CD33 is mainly conveyed by upregulation of downstream pathways. Rosen et al. could show that the cytotoxicity by calicheamicin is not sufficient to induce apoptosis. GO resistant cell lines showed upregulation of the important pro-proliferative PI3K pathway and the application of an AKT inhibitor was able to overcome acquired GO resistance in vitro [91]. GO resistance is therefore mainly caused by polymorphisms of the binding site, increased degradation of CD33 and activation of pro-proliferative pathways (Fig. 3).

## Resistance to Venetoclax

Venetoclax is a BH3 mimetic, which acts as a BCL-2 inhibitor and induces apoptosis. It is widely used as first and second line therapy for CLL, and in combination therapies in AML. The combination of HMA with venetoclax in patients unfit for conventional chemotherapy has significantly improved the outcome of these patients [92].

However, 34% of newly diagnosed AML patients do not respond to the combination of venetoclax with azacitidine and all patients with an initial response will eventually relapse [92].

A resistance mechanism which is commonly found in CLL patients treated with venetoclax is a missense mutation G101V in *BCL-2*. It modifies the binding site of venetoclax [93]. However, this mechanism has so far not been observed in AML patients.

Retrospective analyses of patients who participated in the registration trials identified molecular markers that are associated with response to venetoclax.

Better response rates were seen in patients with *NPM1* or *IDH2* mutations, whereas at relapse selection of *FLT3-*mutated clones could be observed [94]. A deeper analysis showed that relapse in patients could not be pinpointed to a single mechanism, but rather the activation of several different pathways like kinase signalling, RNA-splicing, epigenetic modification, transcription and tumour suppression [95]. It is unclear whether the emerging aberrations are induced by the treatment or are selected from a pre-existing clone due to the selection pressure under treatment.

One central causal mechanism of acquired resistance to venetoclax treatment is the circumvention of pro-apoptotic signalling via BH3-mimetics. Several studies could show that BCL-XL and MCL-1 as homologues of BCL-2 can fulfil the same anti-apoptotic role as BCL-2, but are not inhibited by venetoclax [96–98]. Accordingly, blasts showing a monocytic phenotype were resistant to venetoclax as they—in analogy to healthy monocytes—depend on MCL-1 instead of BCL-2. Therefore, at relapse there is often a dominant monocytic clone [99]. Based on these findings, the in vitro use of MCL-1 inhibitors is able to overcome resistance to venetoclax [100, 101]. Other components of apoptosis may be associated with venetoclax resistance such as TP53, BAX, BAK, PMAIP1 and TFDP1 [102].

When looking at venetoclax resistance, it is important to note that the molecule does not only inhibit BCL-2, but the combination with hypomethylating agents also leads to a decrease in amino acid uptake that diminishes the cellular energy source [103, 104]. To overcome this inhibition leukemic cells activate fatty acid metabolism by upregulating several genes of this pathway [105]. Another way to recruit additional energy sources is an increased production of NADP + [106]. Thus, upregulating alternative pathways of energy resources seems to be a potential mechanism for acquired resistance to venetoclax [105, 106].

**Fig. 4:**
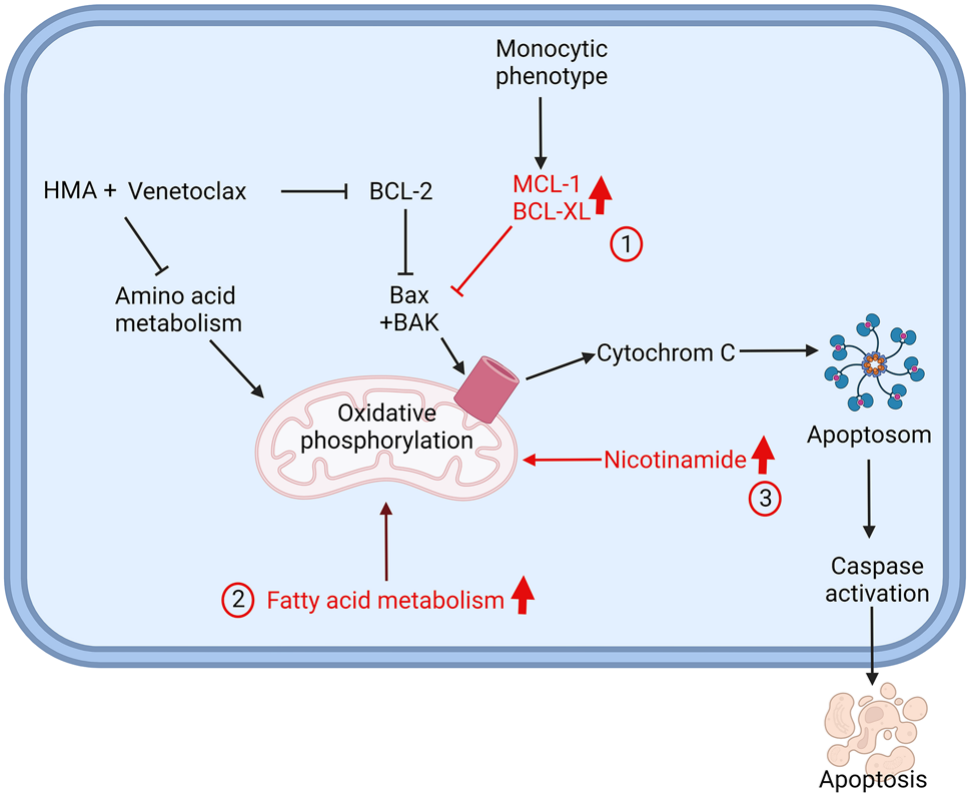
Overview of resistance mechanisms against venetoclax. Venetoclax is a BH3 mimetic, which inhibits BCL-2, an inhibitor of apoptosis. Common resistance mechanisms are: 1: Upregulation of MCL-1 or BCL-XL; 2: Upregulation of fatty acid oxidation; 3: Upregulation of nicotinamide leading to increased NADP + levels. (Created with BioRender.com)

In summary, primary resistance to venetoclax is associated with variable, incompletely understood changes of cellular biology with high inter-patient variability. Acquired resistance to venetoclax mainly emerges by circumventing the pathways, which are inhibited by venetoclax, namely BCL-2 and amino acid metabolism (Fig. 4).

## Outlook: resistance to immunotherapy

In contrast to lymphoid malignancies, immunotherapy in AML is still facing many challenges. The bone marrow niche of AML provides an immunosuppressive environment protecting the leukemic cell from immune attack. For example, regulatory T-cells and myeloid derived suppressor cells are increased in the bone marrow niche [107, 108]. However, pathways involving the immune response appear to be vital for pathogenesis of AML and a valuable therapeutic target. Relapse after stem cell transplantation is in most cases not associated with new mutations in proliferative pathways, but with changes to the immune response. Patients with relapse after alloHSCT may show a downregulation of HLA class II expression and therefore fail to properly present antigens [109]. The importance of the immune system and its therapeutic potential is also underscored by the role of the graft-versus-leukemia-effect and the ability of donor lymphocyte infusions (DLI) to boost the graft vs leukemia effect as an important pillar of treating post-transplant relapses. Based on this central role of the immune response in relapse after alloHSCT, the application of ipilimumab in patients with relapse after alloHSCT activated T-cells and led to remission in some patients [110]. Other checkpoint inhibitors such as nivolumab and pembrolizumab are evaluated in combination with conventional chemotherapy [111, 112]. However, all these approaches have to overcome the immunosuppressive bone marrow environment and—for patients after transplantation—the higher risk of GvHD.

A novel mechanism targeting the immune system is the activation of macrophages. Several CD47-antibodies are being evaluated. CD47 is overexpressed in many cancer entities and acts as a signal to prevent phagocytosis by macrophages [113]. In a phase Ib study, the combination of the CD47-antibody magrolimab with azacitidine was able to induce a response in 64% of the patients, reaching CR/CRi in about 50% of the patients with similar response rates in patients with or without *TP53* mutations [114]. Based on these promising results magrolimab is currently being evaluated in several clinical studies in AML patients.

Although resistance mechanisms to magrolimab have not been described so far, based on its mechanism of action one could consider several ways of resistance. One regulatory point may be the expression of CD47 on the cell surface. CD47 expression is regulated by a number of cytokines and pro-inflammatory mediators on hematopoietic stem cells (HSC), thus enabling migration from the bone marrow to the site of inflammation [115]. This mechanism may also apply to LSCs, as signals from the bone marrow niche may be able to considerably downregulate CD47 expression. CD47 can be located on the cell surface or in the membrane of the endoplasmic reticulum (ER). The localisation is determined by the length of the 3’ UTR: mRNA with a long 3’UTR encodes for CD47 located in the cell membrane, so the amount of CD47 on the cell surface can be regulated at a posttranscriptional level [116].

Binding between CD47 and the SIRPα-receptor on the macrophages is only possible, when there is polyglutamate added at the binding site via the glutaminyl-peptide cyclotransferase like protein [117]. Thus, any interference with binding of the antibody to its target may induce resistance.

## Conclusion

Resistance to targeted therapies is either primary or secondary due to acquired mutations under the selective pressure of the treatment. Although the targets are different, there are some common mechanisms to resistance: On the one hand, there may be mutations at the binding site of the target protein; on the other hand, alternative pathways may be activated to allow cell proliferation and survival. Knowledge of common resistance mechanisms can help to develop strategies that may prevent resistance. For example, the activation of the MAPK-pathway is a common finding. However, currently available MEK inhibitors have limited activity in AML patients and are associated with considerable hematologic toxicity [118–120]. MCL-1- or BCL-XL-inhibition as an add-on to BCL-2-inhibition may be an option to overcome venetoclax resistance. On the other hand, added toxicity may be of concern and requires careful evaluation. Table 2 summarizes current knowledge on possible ways to overcome resistance. Most findings, however, are still limited to in vitro or mouse experiments. Clinical data on sequential therapies is scarce.

**Table 2:** Known therapeutic strategies to overcome resistance to targeted therapies. Mainly evidence is restricted to findings in vitro or mice

Mutation	Resistant drug	Theoretical treatment option	Clinical experience	References
Flt3 on-target mutations	FLT3 inhibitor	Sequential use of FLT3i F691L confers resistance to all known FLT3i	Exposure to another FLT3i after midostaurin induced CR in 29%, FLT3i after quizartinib induced CR in 20%; quizartinib after sorafenib induced CR in 25%, no experience with second FLT3i after gilteritinib	[57]
Upregulation of soluble downstream factors	FLT3 inhibitor	FGF2 inhibitor CXCR4 inhibitor	In vitro evidence only for FGF2 inhibition CXCR4 inhibitor AMD3465 in combination with cytarabine eliminated leukemic blasts in mice	[44, 46]
Upregulation of downstream-pathways	FLT3 inhibitor	AXL inhibitors/ gilteritinib STAT5 inhibitors PIM inhibitor JAK inhibitor	The AXL inhibitor TP-0903 showed prolonged survival in combination with FLT3 inhibition in mice In vitro evidence only for PIM and JAK inhibitors	[31, 47, 50–52]
Increased intracellular pH	FLT3 inhibitor	NHE1 inhibitor	In vitro evidence and engraftment experiments in mice only	[53]
Upregulation of FLT3-ligand	Flt3 inhibitor	Flt3 inhibitor with higher plasma levels, higher affinity to Flt3	Not known	[42]
Binding site polymorphism	GO	Not known		
Increased drug degradation	GO	Not known		
Upregulation of PI3K-pathways	GO	AKT-inhibitor	In vitro evidence only	[91]
Mutation at binding site	IDH1/2 inhibitors	Not known		
Isotype switch	IDH1/2 inhibitors	Ivosidenib after enasidenib; Enasidenib after ivosidenib; Bivalent inhibitors e.g. Ly3410738	Clinical studies ongoing	[74, 75]
Upregulation of downstream pathways	IDH1/2 inhibitors	RAS inhibitor	Not known	[69]
Alternative energy sources	IDH1/2 inhibitors	OXPHOS inhibitor	Effect of combined IDH and OXPHOS inhibition in mice	[76]
Increased expression of MCL-1 or BCL-XL	Venetoclax	MCL-1 or BCL-XL inhibitors	In vitro evidence only	[101]
Utilization of alternative energy sources such as fatty acid metabolism/NADP +	Venetoclax	Inhibition of FAO	In vitro evidence only	[105]

By using targeted therapies, resistant clones will have a proliferative advantage over sensitive clones and inhibiting one clone may not prevent other clones from expansion. Therefore, the sequential use of therapeutics that target different pathways may be an approach to overcome resistance. In addition, the dysregulation of the immune system offers a novel therapeutic area, which needs to be explored in much more detail to derive benefit of immunotherapy for AML patients.
